# Characterization of Mosquito Breeding Sites in and in the Vicinity of Tigray Microdams

**DOI:** 10.4314/ejhs.v21i1.69045

**Published:** 2011-03

**Authors:** Tadesse Dejenie, Mekonnen Yohannes, Tsehaye Assmelash

**Affiliations:** 1 Department of Biology, College of Natural and Computational Sciences, Mekelle University, P.O. Box 231, Mekelle, Ethiopia (*taddej2002@gmail.com); 2 Department of Microbiology, Immunology and Parasitology, College of Health Sciences, Mekelle University, PO Box 1871, Mekelle, Ethiopia

**Keywords:** Anopheles, breeding, characteristics, Culex, larvae, mosquito, Northern Ethiopia

## Abstract

**Background:**

Malaria vector control in Ethiopia has a history of more than 50 years, but malaria remains a major cause of morbidity and mortality in Ethiopia. Thus, targeting the control program on the larval stage is of paramount importance. This study aimed to characterize the aquatic habitats of vector mosquito larvae associated with micro-dams.

**Methods:**

Cross-sectional larval survey was conducted on six micro-dams in Tigray, Northern Ethiopia in 2005/06. The study area on each dam was divided into eight zones. Immature stages of mosquitoes were collected using standard dippers. The physico-chemical characteristics of the aquatic habitats were measured onsite.

**Results:**

A total of 301 aquatic habitats were surveyed for mosquito larvae; in 32.56% (n=301) only Anopheles, in 27.91% only Culex, both genera were found mixed in 21.59% and no mosquito larvae were found in 17.94%. The findings depicted that dissolved oxygen (r = 0.34, p =0.04), pH (r = 0.35, p =0.03), conductivity (r = 0.36, p =0.03), vegetation (F = 3.54, p =0.002), microhabitat (F = 2.65, p =0.04), fauna and bottom surface of the water body were positively associated and important in explaining the presence and abundance of Culex. On the other hand, dissolved oxygen (r = 0.39, p =0.02), pH (r = 0.42, p =0.008), vegetation (F = 5.6, p =0.000), water transparency (F = 2.72, p =0.00), rainfall (F = 2.22, p = 0.027) and fauna were positively associated and important in explaining the presence and abundance of Anopheles.

**Conclusion:**

The findings of this study suggest that both biotic (vegetation and fauna) and abiotic (chemical and physical) factors play a significant role in larvae's habitat preference in both Culex and Anopheles.

## Introduction

The reservoirs formed by dams provide large areas of stagnant water. Presently in Tigrary, there are more than 70 reservoirs with carrying capacity ranging from 50,000 to 4,500,000 m^3^ (1, 2) and the majority of the dams are situated near human settlements at an altitude range of 1700–2700m. The collected water due to such impoundment of dams could result in water-related illnesses, which are transmitted by vectors that live and breed in or around water. Thus, as a potential negative side-effect, dams may create conducive environment for breeding sites of malaria vectors.

Malaria control in Ethiopia has a history of more than 50 years, but still now, malaria is a major cause of morbidity and mortality in Ethiopia (26). Current control strategies are institution-based diagnosis and treatment, presumptive treatment by community health workers (CHWs), selective indoor residual spraying, killing of larvae by pesticides in selected urban areas, and epidemic control. In spite of these control efforts, malaria remains a major public health problem in Ethiopia (26). A study done in kenya, indicated the phyicco-chemical characteristics such as habitat type, floating debris and emergent plants were found to be the key factors determining the presence of *Anopheles* larvae in the habitats (22).

The oviposition preferences of adult females and the ability of immature stages of mosquito to both biotic and abiotic environmental conditions of a given aquatic habitats determine the abundance and distribution of mosquito larvae (23). Therefore, characterizing mosquito breeding sites in order to determine their influence on anopheline and culicine distribution and densities could be very useful to understand the variations observed in malaria transmission intensity and to plan more efficient vector control strategies. However, no substantial information exists on the influence of breeding sites distribution, environmental and physicochemical characteristics on malaria vectors distribution and densities in Tigray, Northern Ethiopia.

For effective larval control, the type of habitat should be considered and most productive habitat type be given a priority in the mosquito abatement program. It has long been argued that the best way to reduce malaria transmission is to target adult female mosquitoes with insecticides that can reduce vector density, longevity and human feeding frequency (3, 4, 5, 6). However, these analyses have ignored a fundamental biological difference between mosquito adults and the immature stages that precede them. Adults are highly mobile, flying insects, which can readily detect and avoid many intervention measures, whereas mosquito eggs, larvae and pupae are confined within relatively small aquatic habitats and cannot readily escape control measures (7).

The establishment of artificial water bodies to enhance agricultural development has a history of facilitating changes in the frequency and transmission dynamics of malaria in the lowland below 1900m (8), but analyses of these environmental risk factors are limited. Generally, mosquito larvae are filter feeders of organic particulates; they specifically feed on algae, bacteria and other microorganisms (24). Mosquito larvae feed mainly on most carbohydrates and their products, animal proteins, yeast, infusion and other food sources (25).

Knowledge on where mosquitoes breed and why they prefer certain water bodies over others is very important for sound mosquito control strategies (9). However, our understanding of anopheline larval ecology is limited, and the knowledge is insufficient to achieve effective vector control through the means of larval control (10). For example, it is unknown what causes vector abundance and distribution, and how the mosquito larval abundance is regulated in the diverse aquatic habitats. Focusing efforts to larvae, however, requires sound knowledge of the local situation and the behavior of the vector population. A basic understanding of the aquatic stages of vectors would be extremely relevant for malaria control (11). Thus, the environmental factors associated with the breeding sites of mosquito larvae in the six selected micro dams in Tigray were studied.

The objectives of this study were: to assess the presence and abundance of mosquito larvae in and around the dams and also to evaluate the associations between the characteristics of the water bodies that influence the abundance of anopheline and culicine mosquito vectors. This assessment would provide the necessary data related to the characterization of breeding sites and habits of the resident species of mosquitoes. It would further give data which could help to determine spatial distribution and to characterize the larval habitats and associated environmental parameters that influence the abundance of *Anopheles* mosquitoes.

## Materials and Methods

**Figure 1:**
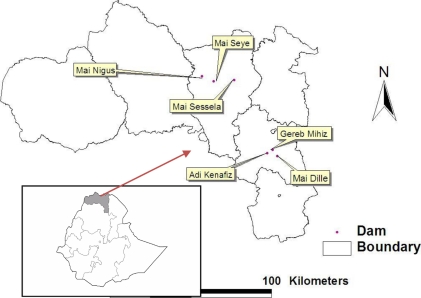
Map of study area

**Geographical Location of the Dams:** Mai Nigus, Mai Sessea and Mai Seye dams are located in the Central Zone of Tigray while the other three study dams (Mai dille, Adi Kenafiz and Gira Bered) are located in the Southern Zone of the Regional State of Tigray (Figure 1).

There might be a year-to-year variation in relation to variation in precipitation in a given locality, and this may interfere with this approach. Whatever approach taken, there might be uncertainties, thus to compensate such problems, there should be sufficient replication. A design which largely involved a spatial and temporal approach was employed. Our design involved largely a spatial and temporal approach. Thus, six dams and nearby water bodies were selected; we selected 3 dams from Central Tigray and three from South Tigray on the basis of the history of mosquito and malaria, and the altitude is greater than 1900 and less than 2100masl.

**Figure 2:**
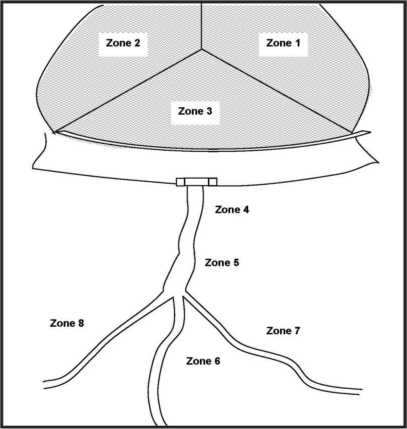
Division of the dam area into several zones or grids as specific sites for mosquito larvae sampling

After selecting the dams, each was subdivided into eight zones or grids. The sites were classified into three main divisions: dam area right side (zone 1), left side (zone 2) and base of impoundment (zone 3); downstream valleys (zone 4–6) and irrigation sites (right side zone 7 and left side zone 8) see (Figure 2). Then, successive larval samples were taken from the sites. The first larval sample was collected in November 2005, the next in February 2006, April 2006, May 2006 and the last in September 2006. A total of 6 dams and 301 potential breeding sites (according to Figure 2) were examined for mosquito larvae.

The type of aquatic habitat, its size and physico-chemical conditions, as well as density and developmental stage of the *Anopheles* larvae were recorded in a data sheet. The densities were calculated as number of mosquito larvae per dip (350ml volume). According to the size of a site, a representative number of dips were taken. Using standard dipper (350ml), a minimum of 6 and a maximum of 60 dips were taken from each type of water body. Sampling was proportional to surface area of the different types of habitat. Six dips were taken within a focal habitat within a 10 m2 area or every 10m, when the habitat is linear. Dipping takes place where larvae are expected (edges of sites, around vegetation, shallow areas, etc). From each collection site, mosquito larvae were transferred into separate vials, killed by gently heating and preserved in 70% alcohol. All aquatic forms of mosquitoes were counted and identified into genera (12).

The environmental variables, chemical and physical characteristics were measured with respect to larval habitat. The chemical characteristics such as percentage of dissolved oxygen, temperature, pH and conductivity were measured using a WTWA Multi 340 I electrode. Physical characteristics of the mosquito larval habitat conditions, including depth, water transparency (turbidity), vegetation and bottom surface of the habitat, fauna and microhabitat were measured. Turbidity of the water was categorized as clear, turbid or foul, whereas bottom substrate type was classified as muddy, rocky, sandy or combination. On the other hand water depth was measured using a ruler, while vegetation type, which can be seen by a naked eye, was classified 0 = No vegetation, 1 = grass, 2 = herb, 3 = algae, 4 = aquatic vegetation and 5 = dead vegetation).

Univariate and multivariate analyses were performed using the statistical program STATISTICA 9.0. Seasonal dynamics of *Anopheles* and *Culex* larval densities was analyzed by executing repeated measure of ANOVA. Multiple regression analysis was done to explain the variation in *Anopheles* and *Culex* larval densities with respect to physical characteristics (water transparency, vegetation, fauna, microhabitat and nature of bottom surface of the water body). Pearson correlation analysis was also used to assess the relationship between the densities of mosquito larvae and chemical characteristics of the water body. Variables used for multiple regression analysis and Pearson correlation analysis were log transformed (log10 n + 1) prior to statistical analysis. All statistical analyses were performed at the 5 % significance level.

## Results

**Larval mosquito Surveys:** A total of 301 habitats (sites) were surveyed within the altitude range of 2000 to 2070masl. Mosquito larvae were recovered in 82.04% (247/ 301) of the habitats. We found Anopheles larvae in 98 (32.56%), culicines in 84 (27.91%), both genera mixed in 65 (21.59%), and in 54 (17.94%) we did not find larvae of mosquito.

**Figure 3:**
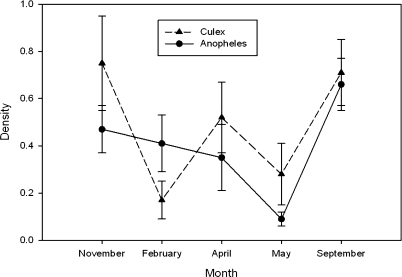
Comparison of the density of *Culex* and *Anopheles* aquatic stage in and near microdams in Tigray from November 2005 – September 2006

A repeated measure of ANOVA showed seasonal dynamics of mosquito larvae in the studied dams. High densities of culicine aquatic stages were observed in September and November, and least in February (F = 4.433 and p < 0.002). The densities of *Anopheles* larvae were highest in September and November, whereas very low density was observed in May. The differences in densities were significant among months (F = 4.423 and p = 0.002) (Figure 3).

**Chemical Characteristics of larval habitat:** The presence/abundance of culicine aquatic stages was closely associated with dissolved oxygen (r = 0.34 and p = 0.04), pH (r = 0.35 and p = 0.03) and conductivity (r = 0.36 and p = 0.03) (Table 2). Occurrence of *Anopheles* larvae was positively associated with dissolved oxygen (r = 0.39 and p = 0.01) and pH (r = 0.42 and p = 0.008). Water temperature was not associated with the occurrence of *Culex* and *Anopheles* (Table 1). Both high and low densities of mosquitoes were found at higher temperatures (p = 0.72 for *Culex* and p= 0.76 for *Anopheles*).

**Table 2:** Results of multiple regressions on Density of Anopheles and Culex larvae in relation to key variables in a set of 6 dams in Tigray in 2005–2006.

Density of *Anopheles*				
R^2^ = 0.18; Adjusted R^2^ = 0.17; F(4, 296) = 16.2; p<.000				
	Beta	Std.Err.	t(296)	p-level
Intercept			2.584	0.010
Vegetation	0.276	0.060	4.602	0.000
W. Transparency	−0.149	0.055	2.720	0.007
Rainfall	0.119	0.054	2.220	0.027
Fauna	0.126	0.060	2.113	0.035
Density of *Culex*				
R^2^ = 0.12; Adjusted R^2^ = 0.094; F(8,292) = 4.9; p<.000				
	Beta	Std.Err.	t(292)	p-level
Intercept			3.058	0.002
Vegetation	0.201	0.066	3.053	0.002
Bottom	−0.158	0.056	−2.804	0.005
Fauna	0.145	0.064	2.282	0.023
M habitat	−0.269	0.130	−2.065	0.040
W. Transparency	−0.103	0.059	−1.763	0.079

**Table 1:** Average values (± SE) and correlation of the chemical variables used to characterize the aquatic habitats in relation to *Culex* and *Anopheles* larvae density

Variable	Mean ± SE	Correlation	
		*Culex*	*Anophelus*
Dissolved oxygen (mg/L)	6.04 ± 0.259	r =0.34, p =0.04	r =0.39, p =0.02
pH	8.34 ± 0.068	r =0.35, p =0.03	r =0.42, p =0.008
Conductivity (µs)	560.33 ± 42.58	r =0.36, p =0.03	r =−0.02, p =0.93
Temperature (°C)	24.26 ± 0.2137	r =−0.05, p =0.72	r =0.06, p =0.76

**Physical Characteristics of larval habitat:** The results of multiple regression analysis showed that vegetation, water transparency, rainfall and fauna were more important in explaining the abundance of *Anopheles* larva than other variables. On the other hand, bottom surface nature and microhabitat were more important in determining the density of *Culex* larvae instead of water transparency (Table 2).

**Figure 4:**
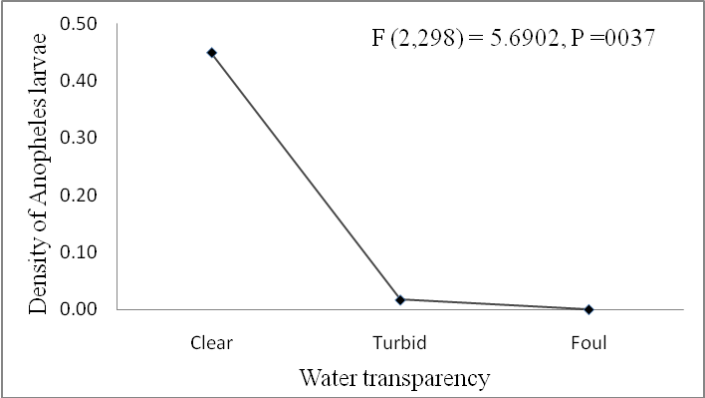
*Anopheles* larval density in relation to the degree of transparency of larval habitats (Clear water, Turbid and Foul water) around six microdams surveyed in Tigray, northern Ethiopia (November 2005 to September 2006.

Water transparency showed a positive association with the density of *Anopheles* larvae (F (2,298) = 5.6902, P =0037) (Table 2 and Figure 4). Yet, it tends to show negative association with density of *Culex* (p>0.079).

Generally, no larvae were found in zone 3 and rarely encountered in zones 1–2 (see Figure 2 for zone arrangement). Even though we found the highest densities of *Anopheles* larvae in zone 8 (the irrigation fields), there was no significant difference in zone preference in *Anopheles* (p>0.05). Unlike *Anopheles*, in *Culex* larvae were not or rarely encountered in the dam structure, but common downstream and in the irrigation canals (p = 0.04, Table 3). Both *Anopheles* and *Culex* showed positive association with vegetation cover. The highest densities of mosquito larvae were found in sites with a combination of grasses and dead plants (Table 2).

## Discussion

More than half of the surveyed water bodies were breeding habitats of mosquito larvae. Besides, the presences of dams create favorable condition for the presence of *Culex* and *Anopheles* all year round. On top of this, the density of *Anopheles* and *Culex* larvae showed seasonal dynamics, which might be associated with the dynamics in rainfall (availability of water). The highest density of mosquito larvae was observed in September 2006, following the long heavy rainy season and lowest in May. In Central Tigray whereby Mai Nigus, Mai Seye and Mai Sessela microdams are found, sampling was done after a short heavy rain. Thus, low density of mosquito larvae in May might be associated with floods, which might wash the eggs and larvae. Other reports also indicated that heavy rains with flooding eliminate *Anopheles* habitats, and thus larvae were rarely encountered during sampling (13).

Both anopheline and culicine larvae were positively associated with dissolved oxygen. Previous reports also indicated similar association of *Culex quinquefasciatus* and *Anopheles arabiensis* larvae with dissolved oxygen (14). Oyewole *et al* (27) support the idea that optimum dissolved oxygen might have contributed for survival and breeding of *Anopheles* larvae. Abundance of *Culex* showed positive association with conductivity, but no association with *Anopheles*. As conductivity is the measurement of the accumulation of ions in a solution, we have no justification why conductivity is positively related with abundance of *Culex*. Unlike our finding, Burke *et al* (15) reported negative association of conductivity with *Culex quinquefasciatus* larval presence.

Almost all our study dams were alkaline (pH >7) and both anopheline and culicine larvae were also positively associated with pH. Thus, our findings disagree with Adebote *et al* (16), which reported the preference of anopheline species to low pH values, acidic nature. On the other hand our findings agree with the positive association of culicines which have greater range of pH values (pH 5.86 – 9.85) (16). On the other hand, both *Anopheles* and *Culex* did not show any association with water temperature. Similar results were obtained by McCrae (20) and Stephen (17). These results suggest that, factors other than water temperature might have played an important role in egg hatchability. There was no association between water temperature and abundance of mosquito larvae, but in other studies, for example, *Cx. quinquefasciatus* larval survival and development into adult was highest in water temperature between 20 to 30 °C (18). The requirement of this temperature range in which the temperature of our dams is in between this range, thus this could be the reason for not showing insignificant association between temperature and mosquito larva abundance in this survey.

Vegetation was also important predictor for *Anopheles* and *Culex* larvae presence and abundance. Similar positive association was reported for *An. gambiae* larvae (19). The presence of vegetation could help the larvae to hide themselves from their predators. *Anopheles* showed positive association with water transparency. Similar findings with *Culex quinquefasciatus* were reported indicating negative association with total dissolved solids (TDS), which indirectly shows positive association with water transparency (15). Positive association of *Culex quinquefasciatus* with total dissolved solids was also reported by Muturi *et al* (14). Our finding seems to contradict to the reports of Stephen (17) that suggest the preference *An. gambiae* is to turbid water than clear water for oviposition. This could be due to the fact that during the rainy season, *An. arabiensis* favors turbid water, but during the dry season, as there is no or rare turbid waters created by rainwater pools it exist in the clear water.

Abundance of *Anopheles* and *Culex* larvae were positively associated with presence of other aquatic fauna. This might indicate the existence of favorable environment for various aquatic fauna. On the other hand, sandy bottom surfaces showed the highest positive association with *Culex* and the least with rock. Although the specific soil type was not analyzed in this survey, other reports indicated that there is variation in development of *Anopheles* larva among the different soil types (21).

There seems to be a seasonal variation in abundance of mosquito larvae. One remark should be made here: it is likely that small water bodies associated with the dams (such as small ponds, hoof prints of cattle, irrigation channels) rather than the dams themselves host most of the mosquito larvae. The findings of this study also suggest that both biotic (vegetation and fauna) and abiotic (chemical and physical) factors play a significant role in larval habitat preference in both *Culex* and *Anopheles*. Thus, such factors should be considered when designing an integrated vector control program. Further cross-sectional study of the aquatic mosquito larvae breeding and none breading habitats is recommended: including all biotic and abiotic variables using accurate quantitative measurements.
